# Species-free species distribution models describe macroecological properties of protected area networks

**DOI:** 10.1371/journal.pone.0173443

**Published:** 2017-03-16

**Authors:** Jason L. Robinson, James A. Fordyce

**Affiliations:** 1 Illinois Natural History Survey, Prairie Research Institute, University of Illinois, Urbana- Champaign. Champaign IL, United States of America; 2 Department of Ecology and Evolutionary Biology, University of Tennessee, Knoxville, TN, United States of America; Universita degli Studi di Trento, ITALY

## Abstract

Among the greatest challenges facing the conservation of plants and animal species in protected areas are threats from a rapidly changing climate. An altered climate creates both challenges and opportunities for improving the management of protected areas in networks. Increasingly, quantitative tools like species distribution modeling are used to assess the performance of protected areas and predict potential responses to changing climates for groups of species, within a predictive framework. At larger geographic domains and scales, protected area network units have spatial geoclimatic properties that can be described in the gap analysis typically used to measure or aggregate the geographic distributions of species (stacked species distribution models, or S-SDM). We extend the use of species distribution modeling techniques in order to model the climate envelope (or “footprint”) of individual protected areas within a network of protected areas distributed across the 48 conterminous United States and managed by the US National Park System. In our approach we treat each protected area as the geographic range of a hypothetical endemic species, then use MaxEnt and 5 uncorrelated BioClim variables to model the geographic distribution of the climatic envelope associated with each protected area unit (modeling the geographic area of park units as the range of a species). We describe the individual and aggregated climate envelopes predicted by a large network of 163 protected areas and briefly illustrate how macroecological measures of geodiversity can be derived from our analysis of the landscape ecological context of protected areas. To estimate trajectories of change in the temporal distribution of climatic features within a protected area network, we projected the climate envelopes of protected areas in current conditions onto a dataset of predicted future climatic conditions. Our results suggest that the climate envelopes of some parks may be locally unique or have narrow geographic distributions, and are thus prone to future shifts away from the climatic conditions in these parks in current climates. In other cases, some parks are broadly similar to large geographic regions surrounding the park or have climatic envelopes that may persist into near-term climate change. Larger parks predict larger climatic envelopes, in current conditions, but on average the predicted area of climate envelopes are smaller in our single future conditions scenario. Individual units in a protected area network may vary in the potential for climate adaptation, and adaptive management strategies for the network should account for the landscape contexts of the geodiversity or climate diversity within individual units. Conservation strategies, including maintaining connectivity, assessing the feasibility of assisted migration and other landscape restoration or enhancements can be optimized using analysis methods to assess the spatial properties of protected area networks in biogeographic and macroecological contexts.

## Introduction

The anticipation of ecological impacts, as a consequence of climate change, has hastened many conceptual and analytical developments in ecology and biogeography. For example, the rapid development and dissemination of climate data has facilitated widespread efforts to predict past, current and future patterns of biodiversity [1–2]. The potential for the extinction of plant and animal taxa, under climate change, has provided strong motivation for conservation planning [3–9]. As climates shift away from current conditions, the distribution of many species are certain to change in response [10–12] and are likely to continue changing [8].

No matter how or what ecological mechanisms, climate features or other contingent limiting factors are ultimately found to influence populations of plant and animal species, management decisions forced by climate change can be informed by predictions of the fate of climate features on the landscape independently of the peculiarities of particular species or taxonomic groups [13–14]. For example, tourism patterns associated with climate features in snow skiing regions may be altered by changing climate patterns [15,4], with cascading effects on economic drivers of local or regional human communities and governments. Geographic features, including climate or ecological features, are non-randomly distributed across the landscape [16–17] which is one reason why they can be such efficient correlates of ecological regions and species distributions [18]. As a paradigm for landscape ecology, the distance-decay of similarity of geoclimatic and ecological features is a fundamental principle for framing our understanding of the ecological patterns that emerge from spatially autocorrelated distributions of individuals [19]. Tobler’s Law [16] succinctly captures this idea: “everything is related to everything else, but near things are more related than distant things”.

Species distribution models (SDMs) utilize Tobler’s Law by estimating species-environment relationships from observed occurrences, and then projecting those relationships onto sets of future conditions [1, 20–23]. These methods not only predict individual species occurrences but may even predict macroecological patterns across large areas or geographic regions in future scenarios [24]. How PAN units capture changes in these patterns is to some extent a function of how individual units occupy spatial gradients of environmental similarity, ultimately capturing different ecoregions or ecosystems. No matter what causal relationships link species occurrences to the predictor variables used for SDM, protected areas in a network are fixed samples from whatever patterns of background environmental variation happen to exist. As fixed locations, the environmental conditions within protected area boundaries have particular geographic distributions, and the variation of these factors across the landscape or protected area network (PAN) can be informative as a planning and management tool [14, 25–26].

In gap analysis, SDMs may be used to summarize predicted changes in the distribution of species in PANs or other areas, under different climate change scenarios [27]. Gap analyses of protected area “performance” can use occurrence data and interpolated estimates of species ranges [28], or stack individual species distribution model predictions (S-SDM) to estimate species richness and protected area coverage [29–30]. Any predictive method introduces particular types of errors (scale and prediction errors) into an analysis, but S-SDMs can potentially multiply these errors at several different steps. This is particularly problematic for S-SDMs, since assumptions about the causes of observed species-climate relationships, or equiprobable dispersal and transport across an entire study region [17], or indirect area effects [31] and other assumptions on the sources of systematic prediction bias are likely to be unmet [32].

Other methods have been developed to more directly evaluate predicted climate change trajectories for PANs, without the confounding filter of idiosyncratic species distributions (e.g. [33–34]. These approaches have conceptualized individual protected area units as geographic samples from the background distribution of climate states within a region or network of protected areas, but what have not been included with these tools are ways to describe the background context, or spatial patterns of similarity, of geographic areas. The “range-like” properties of protected area boundaries can be exploited to develop climate footprint envelopes of protected areas or measures of the geographic areas occupied by climate states like those occurring within a protected area unit. Such an “envelope” would predict the full extent and geographic distribution of the climate states that occur within a predicted area.

In this paper we outline a method to measure this performance of a protected area network consisting of 163 non-coastal national parks in the 48 contiguous states, by analyzing the coverage and persistence of climate features in this PAN under a model of current conditions and in an example future scenario. Our method complements pre-existing analyses built from S-SDM, and eliminates problems facing the interpretations of those models. We suggest that the climate envelope method is a special case of general SDM or envelope methods, equivalent to a Q-mode raster analysis of the site-environment relationships. The geographic areas we predicted from SDM models built from protected area boundaries are areas *like* species distributions, where each species is endemic to a single protected area in the network. Thus our method is equivalent to an analysis of a protected area network as if each unit is the range of a hypothetical endemic species.

## Materials and methods

### Data

**Fig 1:**
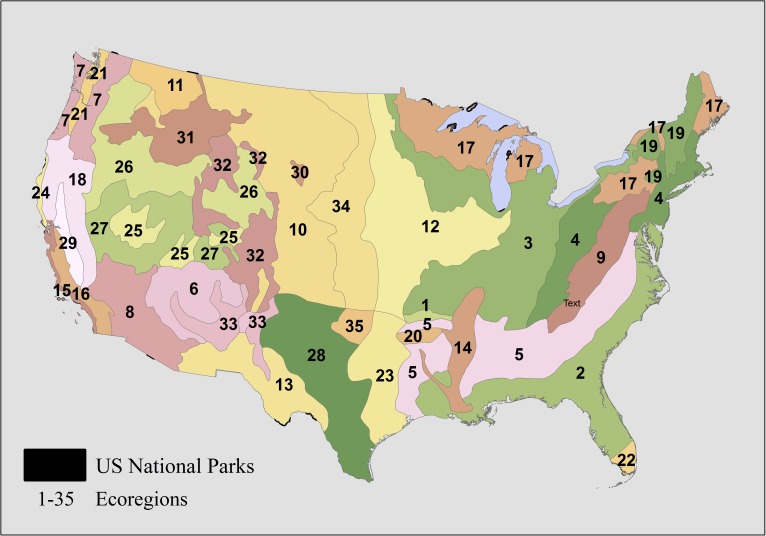
Ecoregions of the US (Bailey 1995) and selected U.S. National Park Service units in the lower 48 states. Numbers correspond to ecoregion codes in S1 Table. Park boundaries slightly exaggerated for illustration.

We obtained GIS [35] shapefiles of the boundaries of national parks from the Office the Assistant Secretary for Research and Technology Bureau of Transportation Statistics (Fig 1). These data are available online at http://www.rita.dot.gov/bts/sites/rita.dot.gov.bts/files/publications/national_transportation_atlas_database/2012/zip/parks.zip, last accessed February 2016. We used a shapefile for the ecoregions of the United States [36], obtained from the National Atlas (Fig 1) to summarize changes in the distributions of climate envelopes in the future scenario. We used five variables from BIOCLIM global temperature and precipitation data for the current [37] and International Center for Tropical Agriculture HADCM3 2050 a2a emissions scenarios [38], downscaled to 1 km^2^ resolution (accessed Aug. 2011). We selected five minimally inter-correlated predictors from the BIOCLIM variables (Annual mean temperature, temperature seasonality, maximum temperature of the warmest month, precipitation of the wettest month and the precipitation of the driest month). All environmental layers and shapefiles were clipped to the extent of the 48 conterminous United States.

### Park climate envelope models

We used MaxEnt [39–40] to fit models of the climate envelope occupied by these national parks. MaxEnt is a machine learning algorithm that uses presence data and “pseudo-absences” drawn from the background to fit parameters to the model in such a way that the final model has the maximum possible entropy among sample points, in environmental space. This approach requires spatial coordinates for occurrences, so we used the GIS to find all the raster centroids within a buffer distance of 2√2 kilometers from park boundaries (the diagonal distance from the centroid to the corner). To eliminate discrepancies between models and local climates, related to lake and ocean effects not captured by global models, we eliminated park cells within 5 km of ocean coastlines. We then eliminated all parks with fewer than 20 raster cells to reduce effects of small sample sizes on model performance [41]. This left 163 national parks which fit our criteria for modeling (names and locations detailed in S1 Table). We used MaxEnt to build models of the climate envelopes of each national parks, using the default options (save one; we increased the number of background points to 100,000) and for each park we used the maximum sensitivity + specificity (MSS) threshold to transform the cumulative output into a binary presence-absence prediction. We summed these predictions to provide a stacked map of the accumulation of park climate envelopes across the study area and ecoregions and to summarize the individual behaviors of PAN units in the network.

### Future projections

To obtain predictions of the fates of these climate envelopes in the future model/emissions scenario, we projected the envelope of each current conditions model onto the future climate scenario, applied the MSS threshold to derive a presence-absence prediction for each envelope, then measured the change in the total area occupied by the envelope of each park, upon the landscape and within the boundary of the associated protected area. Since these parks occur across a large subcontinental region, and across a number of ecologically relevant natural divisions summarized as ecoregions, climate change might interact with the distribution of protected areas and ecoregions such that some ecoregions are more heavily (or lightly) represented within land holdings of this protected area network. We used Geospatial Modelling Environment [42] to summarize the number of climate envelopes predicted within polygons of each ecoregion.

### Statistical tests

We log transformed the square root of the area of parks and climate envelopes to approximate normality, then used linear and logistic regression to explore regression relationships among the properties of the PAN units, climate envelopes and ecoregion boundaries. The extent to which climate envelopes of PAN units occur across the landscape is analogous to classical measures of *richness* of species. Analogs to classical *species-area* relationships can be expressed, for climate envelopes, as the ratio of the predicted climate envelope of a PAN unit and the area of the PAN unit boundary. The extent to which climate envelopes occur across some predetermined area, such as a protected area unit or ecoregion, is analogous to measures of *occupancy* in a species distribution models. Sustained occupancy, in a future scenario, we describe as the *persistence* of a climate envelope (e.g. when a map pixel within a protected area unit is predicted in both scenarios). To consistently calculate occupancy across a PAN described with intricate vector geometries, we used ArcGIS to rasterize the park boundaries to pixels the same scale as the model outputs and then used Geospatial Modelling Environment [42] to summarize the number of predicted pixels in each park. We plotted this occupancy-area relationship for the current and future as a heuristic for assessing the cumulative distribution of PAN unit climates, within protected area boundaries, in the current and future scenarios. We ranked ecoregions on the basis of the proportional representation of PAN unit climate envelopes, assuming that ecoregions with a high proportional area represented within at least one protected area climate envelope to be better buffered to the effects of climate change than ecoregions with very little area predicted by protected area units.

## Results

### Performance: Properties of park climate envelopes

**Fig 2:**
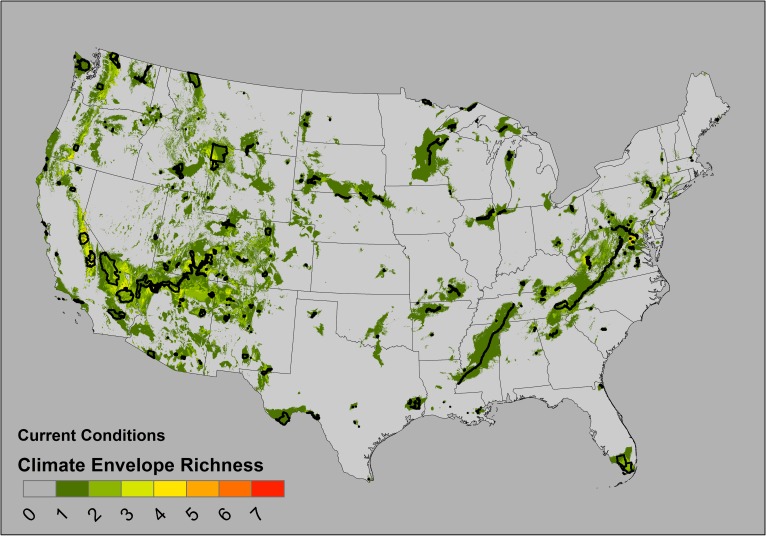
Park climate envelope richness (current conditions). National park boundaries are represented by thin black lines.

**Fig 3:**
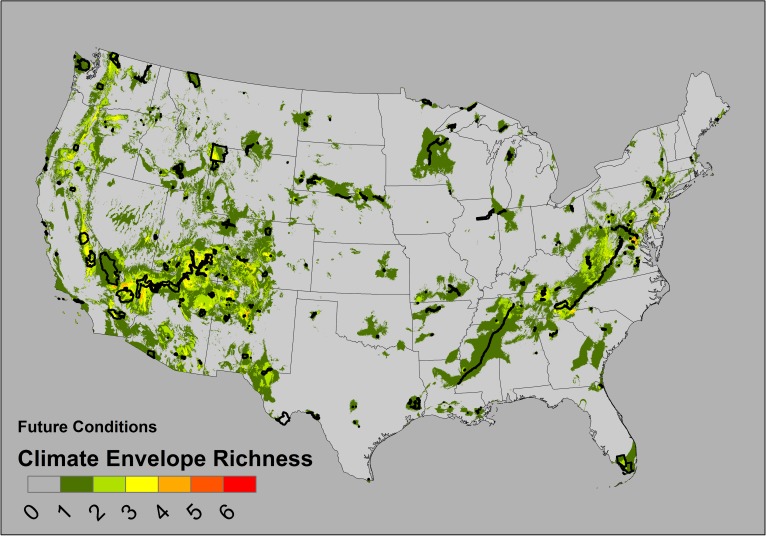
Park climate envelope richness (future conditions). National park boundaries are represented by thin black lines.

**Fig 4:**
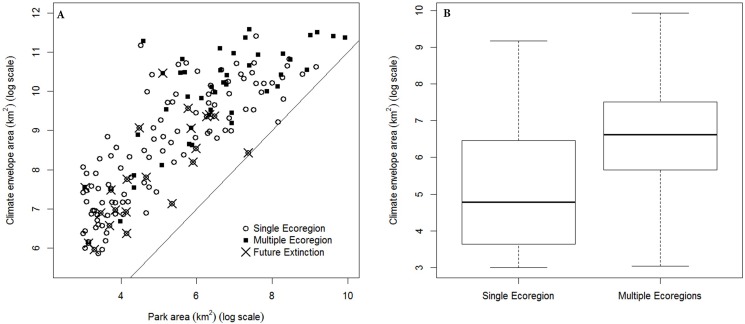
**a**. Predicted climate envelope area as a function of park area (current conditions, log scale), distinguishing protected area climates which go fully extinct from study area in the future scenario. **b.** Parks occurring in only one ecoregion predict smaller climate envelopes than parks in multiple ecoregions (t = -5.15, df = 77.71, p = <0.005).

We modeled the climate envelopes of 163 national parks, ranging from 20 to 20,614 km^2^, with a median unit area of 233 km^2^ (S2 Table). On average, each km^2^ of a park predicted 27.2 km^2^ pixels of area on the map (median) but this ratio ranged from 3–801 among all the 163 parks. In the narrative of our analogy, the potential “range” of a species occurring in every pixel within a park varied with the actual park area by three orders of magnitude. When park climate envelopes were stacked, under current conditions, some map locations accumulate the predicted richness of as many as seven different overlapping climate envelopes (Fig 2). When projected onto 2050 conditions, the general pattern of climate envelope distributions is similar, but predicts lower maximum “richness” or park climate envelopes (no future map cell contained more than six climate envelopes; Fig 3). Larger parks have larger predicted climate envelopes in current conditions (Fig 4A; 95% CI of slope estimate of regression of square root-transformed climate envelope area on square root-transformed park area = 1.99–2.71, F_1, 161_ = 166.7, *p*< 0.0001, *r*^*2*^ = 0.51), but neither park area (square root transformed, F_1, 139_ = 1.737, *p* = 0.19) nor size of the current climate envelope of a park (F_1, 139_ = 3.50, *p* = 0.06, *r*^2^ = 0.018) reliably predicted the area of the future climate envelope. Neither larger parks nor larger current condition climate envelopes predict larger future condition climate envelopes.

### Park climate envelopes in ecoregions

Potential changes in the distribution of climatically similar areas are of interest to managers of protected area networks that protect diverse habitats or areas, or units with particular onsite conservation or management targets. In our study, we used an ecoregion classification as a proxy for the diversity of geoclimatic and ecological features occurring on the landscape. Ecoregions are not equally populated by the units of this PAN (Fig 1; S2 Table). Parks occupying two or three ecoregions predicted larger climate envelopes than parks occurring within a single ecoregion, (Table 1, Fig 4B; t-test on log transformed envelope area, t = -5.15, df = 77.71, p = <0.005) but the climate envelopes of these parks occupying multiple ecoregions are not less likely to go extinct in the future scenario (data in Fig 4A; likelihood ratio test, χ^2^ = 1.98, *p* = 0.37). Large areas of 3 ecoregions are predicted by the climate envelope of at least one park; 2 of these ecoregions are in the southeastern US and potentially share some plant and animal species (Table 1; Everglades and Central Appalachian Broadleaf Forest-Coniferous Forest-Meadow ecoregions).

**Table 1:** Climate envelopes dynamics within ecoregions of the US (Bailey 1995).

Figure Code	Ecoregion	Area	Current Area Not Predicted	Future Area Not Predicted	Future Not Refugia	Current Fraction Not Predicted	Future Fraction Not Predicted	Future Fraction Not Refugia
1	Ozark Broadleaf Forest—Meadow Province	23764	12918	1099	23764	0.544	0.046	1.000
2	Outer Coastal Plain Mixed Forest Province	614697	590692	239634	606098	0.961	0.390	0.986
3	Eastern Broadleaf Forest (Continental) Province	1068602	922601	466347	1E+06	0.863	0.436	0.987
4	Eastern Broadleaf Forest (Oceanic) Province	407999	319481	101875	397278	0.783	0.250	0.974
5	Southeastern Mixed Forest Province	701552	567811	45979	686288	0.809	0.066	0.978
6	Colorado Plateau Semi-Desert Province	280869	85391	129520	245764	0.304	0.461	0.875
7	Cascade Mixed Forest-Coniferous Forest-Alpine Meadow Province	230290	119320	175761	205789	0.518	0.763	0.894
8	American Semi-Desert and Desert Province	320415	131091	201206	289065	0.409	0.628	0.902
9	Central Appalachian Broadleaf Forest-Coniferous Forest-Meadow Province	261224	104204	44871	251740	0.399	0.172	0.964
10	Great Plains-Palouse Dry Steppe Province	1220277	1070018	774594	1E+06	0.877	0.635	0.976
11	Northern Rocky Mountain Forest-Steppe-Coniferous Forest-Alpine Meadow Province	171323	124808	142184	170986	0.728	0.830	0.998
12	Prairie Parkland (Temperate) Province	879193	832930	336720	869730	0.947	0.383	0.989
13	Chihuahuan Semi-Desert Province	302682	235954	229366	288383	0.780	0.758	0.953
14	Lower Mississippi Riverine Forest Province	160886	149471	7424	160879	0.929	0.046	1.000
15	California Coastal Chapparral Forest and Shrub Province	38340	23833	27820	30431	0.622	0.726	0.794
16	California Coastal Range Open Woodland-Shrub-Coniferous Forest-Meadow Province	91847	78416	83258	89060	0.854	0.906	0.970
17	Laurentian Mixed Forest Province	630492	552943	449094	593189	0.877	0.712	0.941
18	Sierran Steppe-Mixed Forest-Coniferous Forest-Alpine Meadow Province	268913	169988	233551	252412	0.632	0.869	0.939
19	Adirondack-New England Mixed Forest-Coniferous Forest-Alpine Meadow Province	183941	180393	149353	183662	0.981	0.812	0.998
20	Ouachita Mixed Forest—Meadow Province	32273	30850	42	30954	0.956	0.001	0.959
21	Pacific Lowland Mixed Forest Province	64448	56123	54423	63290	0.871	0.844	0.982
22	Everglades Province	26117	8380	14114	16941	0.321	0.540	0.649
23	Prairie Parkland (Subtropical) Province	285436	265261	49451	275440	0.929	0.173	0.965
24	California Coastal Steppe-Mixed Forest-Redwood Forest Province	18192	8888	11148	13070	0.489	0.613	0.718
25	Nevada-Utah Mountains-Semi-Desert-Coniferous Forest-Alpine Meadow Province	169517	117500	98327	168006	0.693	0.580	0.991
26	Intermountain Semi-Desert Province	658893	534944	320277	635405	0.812	0.486	0.964
27	Intermountain Semi-Desert and Desert Province	427678	320400	168578	404907	0.749	0.394	0.947
28	Southwest Plateau and Plains Dry Steppe and Shrub Province	575411	564321	365713	572609	0.981	0.636	0.995
29	California Dry Steppe Province	73360	72645	72454	73304	0.990	0.988	0.999
30	Black Hills Coniferous Forest Province	15413	12043	13008	15410	0.781	0.844	1.000
31	Middle Rocky Mountain Steppe-Coniferous Forest-Alpine Meadow Province	351075	251317	177768	343748	0.716	0.506	0.979
32	Southern Rocky Mountain Steppe-Open Woodland-Coniferous Forest-Alpine Meadow Province	408619	215985	184248	347271	0.529	0.451	0.850
33	Arizona-New Mexico Mountains Semi-Desert-Open Woodland-Coniferous Forest-Alpine Meadow Province	184266	114916	133788	177031	0.624	0.726	0.961
34	Great Plains Steppe Province	548558	506427	207336	543239	0.923	0.378	0.990
35	Great Plains Steppe and Shrub Province	64838	63531	0	64838	0.980	0.000	1.000

### Performance: Park occupancy and envelope persistence

**Fig 5:**
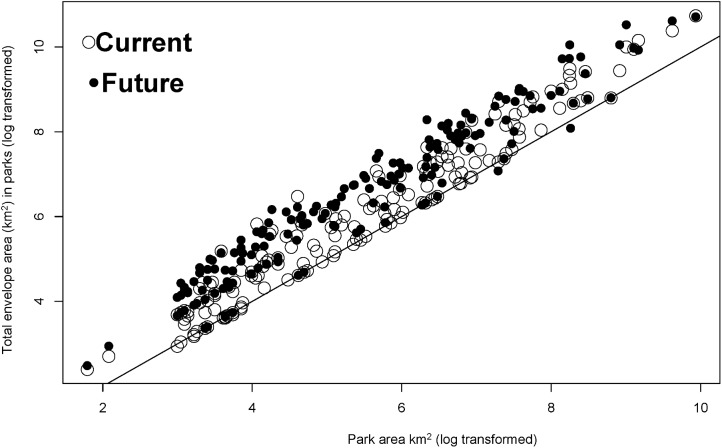
Individual protected area unit occupancy by protected area climate envelopes tends to increase in the future scenario. 1:1 line shown.

These models predict the *landscape context* of protected areas in a network, or some geographic distribution of areas climatically similar to protected areas. This background or context may change with climate change. As one measure of PAN performance, we calculated the ‘occupancy’, or the predicted cumulative sum of all 163 climate envelope “occurrences” in each of the 163 rasterized park boundaries. This measures the occupancy of a PAN unit by all units in the network. In future conditions, larger parks do not systematically predict larger climate envelopes, but larger parks do accumulate more predicted climate envelope occurrences (“occupancy”) from the network than in current conditions (Fig 5). Although the total number of pixels accumulated by parks (with persisting climate envelopes) increases in the future scenario, 77 of these park climate envelopes go extinct *within the boundaries of the units in the network*, in that same scenario. However, only 22 park climate envelopes go extinct from the entire study area (S2 Table). Climate states currently occurring within parks may not occur within those parks in the future, even as they are more broadly distributed across the map, implying that park climates will be less unique (relative to local landscapes) in the future scenario. This predicted future homogenization and higher PAN unit occupancy occurs across the network even as total predicted climate footprint richness in any single park decreases (Fig 3).

## Discussion

### What good is a climate envelope anyway?

Even if we knew nothing at all about the species that occur in a PAN, or the processes by which these occurrences are maintained, our models of the climate footprint of these units retain empirical relations among protected area units and the background explanatory variables. One way to understand those relations is by examining the similarity of a place of interest to the areas adjacent to that place. Tobler’s Law, “Everything is related to everything else, but near things are more related than distant things” [16], suggests that we should expect PANs with many small, heterogeneous or spatially clustered units to differentially capture the landscape-scale environmental variation than PANs where parks are very large or environmentally homogenous. The methods we illustrate here can be leveraged to assist protected area managers actively implementing climate mitigation and adaptation, or other conservation strategies, across different areas in protected area networks.

Previous workers have approached the problem of how PAN units capture climate diversity, or how those climates are predicted to change, by asking how patterns of temperature and precipitation values may change across PAN units occupying environmental gradients. Among [29, 33, 34], all three approaches used climate data to model landscape scale changes in temperature/ precipitation and examined patterns of change within PAN units, leaving aside any predicted effects on the predicted distribution of species within those PANs. In contrast, here we have used the relationship of those variables within each PAN units to build predictive models of the distribution of that specific climate feature on the landscape, as if it were the distribution of some hypothetical species.

Using climate data and S-SDM to interpret predicted changes in species distributions or PAN performance in future scenarios requires a mechanistic understanding of how climate features materially influence the biology of populations or individual organisms. These inferences are necessarily limited in explanatory scope and empirical domain, and there is often a long and tenuous chain of reasoning from S-SDMs to management decisions. Even in the perfect case, when some SDM accurately predicts an entire species distribution across a region, our conclusions about the potentially occupied “niche” of that species are still likely to be confounded by contingencies like local ecological and evolutionary processes [22, 43–44]. The factors that affect the presence or absence from some species at some location are thus probably not direct relationships with climate state predictor variables, so inferences on the ecological or evolutionary traits of modeled species, through the opaque prism of niche estimates, can raise many conceptual and methodological issues [26,43,45–47].

However easy it is to criticize some model; a much greater challenge is to build a better model. In the case of protected area ecological or conservation performance, we are suggesting that (at some size and scale) protected area network units may be “range-like”. One way to conceptualize this is as “large enough to hypothetically contain the known geographic area of some species“. In our study PAN, which is large and varied, some species are apparently endemic to a PAN unit. For example (among other plants and animals), two caddisfly species are each only known from the Great Smoky Mountains National Park (the uenoid *Neophylax kolodskii* Parker, and goerid *Goerita flinti* Parker), and from very few locations within [48–50]. Our best estimates of the distribution of these species, from occurrence data, are limited to a small number of streams, within the same watersheds, in a small region of the park. The climate envelope of this park then would also predict the “range” of these park endemic species (even if it might over-predict the actual area containing these populations) so the analogy of a PAN unit endemic is realistic, for at least some PAN unit sizes. The “range-like” property of the climate envelope of PAN units is a simple and intuitive spatial generalization from traditional gap analysis and species distribution modeling.

Predictive models of the “footprint” of climatic properties of a protected area are intuitively easy to interpret, as “range-like” analogs of species distributions, since each may be built from the same predictor variables. Climate envelopes and SDMs both offer the prospect of within- and among-group comparisons that form the basis of management and conservation decisions in PANs, so to the extent that climate or other modeled features determine species distributions within a network, climate envelope models may be directly biologically relevant to existing management objectives. Using larger areas (e.g. ecoregions) to summarize the behavior of climate envelopes across a protected area network is a novel extension of gap analysis methods to conservation.

#### Q-mode biogeography: Raster maps are “sites” and “species” matrices

With the rapidly burgeoning availability of raster and polygon data on species occurrences, climate, land cover, and protected areas, a litany of interesting predictor variables may now be derived for S-SDM analyses. Rasters are essentially sites-environment matrices, and thus can be analyzed using methods developed for sites-species matrices (including those developed for S-SDM). “R-mode” sites-species matrices have a long history of use in ecology, framing questions about the patterns or processes driving the divergence of the assemblages of plants and animals in areas (e.g., islands), and in particular as examples for how to derive null hypotheses from a background distribution of species among sites [51–52]. Interestingly, [53] considered the value of “Q-mode” analyses (viz. flipping the sites-species matrix 90° to consider as a species-sites matrix), essentially a conceptual predecessor of our analyses of resources occurring within sites. However, uses of MaxEnt or other predictive SDM to model properties of sites *qua* taxa have, to our knowledge, not been published in the ecological or conservation literature.

Protected area networks, as in our example, may protect large areas of land. Land areas will always have *some* climate conditions (and other spatial properties) in any circumstance. Since every site has some climate, values within protected areas will tend to be spatially autocorrelated [16]. The Q-mode analysis of the sites-environment matrix uniquely leverages this autocorrelation (relative to species-based SDMs), since protected area environments are non-random samples from the background distribution of environmental features, and because we are likely to have relatively greater predictive certainty around future environmental conditions than our certainty of how individual species will respond to those projected conditions.

### The landscape context of protected area climates is a conservation target

Given some matrix of species, environmental variables, and a spatial network of individual protected areas, the potential conservation value of the lands within that network can be described by measures of the distribution of the values of climatic explanatory variables, or the “footprint” of these network resources, against the background [54–57]. “Coarse-filter” geodiversity relationships can be used to describe features or properties independently of what species currently occur in the PAN [56]. Because these features tend to be spatially autocorrelated (i.e. follow Tobler’s Law), any protected area will be somewhat similar to the surrounding landscape. The geographic extent of this similarity will likely be unique for each park and landscape, but these properties are extremely relevant to both the ecological processes within the park and the management of those ecological processes in networks of protected areas.

Much recent research in predictive biogeography and conservation has been directed towards the “envelope” occupied by species, underemphasizing the corollary result that protected areas will also tend to have “envelopes” in the same resource space that predicts species presences. Although we are frequently interested in predicting how organisms respond to these features, we emphasize that the locations of protected areas are simply spatially autocorrelated samples from the background distribution of the features that determine species distributions. No matter which is the focus, the aggregate behavior of these individual envelopes can be summarized by metrics of stacked species distribution models (S-SDM) to provide an intuitive framework for understanding the properties of PANs: occupancy, richness, geographic changes in rates of diversity associated with change. The climate envelopes of a protected area network estimate the distribution of climate states conserved by the default boundaries of protected areas, without biases introduced by the contingent processes of ecology, evolution and demographics that shape the actual geographic ranges of individual species.

Gap analyses of S-SDMs are central to many studies analyzing the geographic range or climate envelopes of species in protected area networks (e.g. [58–61] and many more throughout the literature). In the example we presented here, S-SDM of the climate envelopes of each national park are analogous to the hypothetical case of an endemic species with a geographic distribution entirely restricted to within the boundaries of the protected area. The properties of the climate occurring within the protected areas can be used to evaluate several different metrics of PAN ecological performance, without knowledge of the particular biology of individual species within the PAN, a strength of coarse-filter approaches [62].

### Protected area effectiveness: Composition, rarity and future quality

A number of measures and methods have been proposed to measure the value of different aspects of the ecological performance of protected areas. [63] argued that four main categories of performance are generally valued in protected area network units: the *composition* of plant or animal assemblages, the *rarity* of individual elements (either within the PAN or range wide among species) the *future quality* of the habitats within the PAN in some future state and whether the PANs solve *problems* identified by end users or shareholders. The taxon-specific utility of some of these measures of performance may further be limited by gaps in the knowledge of the ecology of organisms or habitats [64]. These concepts are directly applicable to the climate envelopes analysis we have derived here, treating the climates within PANs as “species”.

Here, the *composition* of the conserved group is the aggregate of the individual climate envelopes of each individual protected area unit, which we have described as climate envelope “richness” (Figs 2 and 3). These maps of envelope richness identify geographic areas where there are climates similar to the climate of some protected areas, as well as areas with climates not similar to any PAN unit. Maps will provide intuitive summaries of the distribution of climate envelopes of PAN units, at larger geographic scales. To the extent that ecological communities are determined by climate, protected area climate distributions could serve as an index of environmental redundancy or under-representation within the network [65]. In our example (using the US National Park System), climates occurring within these protected area units tend to be clustered around clusters of protected area units. In regions with numerous parks, the climates occurring within a park may be similar to the climates of several nearby parks. When PAN units are close to each other, units with similar climate envelopes might have similar management concerns, contain similar assemblages of plant and animal species, or respond similarly to climate change. If these units are ecologically connected by migration and gene flow, or by relatively undisturbed landscapes, we would predict increased similarity of species assemblages among those units *even if we know nothing else about the assemblages within these units*. Alternatively, protected area units that are isolated from other units, may have ecological assemblages not well represented across the network. In our example, at the landscape of the conterminous United States, the *composition* of the climates within the protected area system alone is not sufficient to conserve all of climate variability that exists in the larger landscape, since such large regions of the map are not similar to the climates within in any PAN unit (Fig 2) and our PAN does not capture large fractions of many ecoregions (Table 1).

Results from climate envelope analyses can be used to summarize the composition of the envelopes predicted within protected area units themselves, or in larger geographic units like ecoregions. To the extent that ecological communities are shaped by climate, the composition of protected area climates might serve as a measure of environmental redundancy or under-representation within the network [65]. Landscapes in neighboring PAN units, predicting similar climate envelopes, may have similar management concerns, contain similar assemblages of plant and animal species, or respond similarly to climate change. When considered across the landscape of the conterminous United States, the *composition* of the climates within the protected area system alone is clearly not sufficient to conserve all of climate variability that exists in the larger landscape (Fig 1; Table 1). Some ecoregions are simply better represented within this PAN than others (e.g. eastern broadleaf forests), and this will have real material consequences for the management of those PAN units for particular ecological assemblages or populations of individual species. When species occurrence data are limited, as is often the case for broad taxonomic groups [64], these analyses may provide meaningful results to managers that would otherwise be unattainable without intensive inventories and literature searches.

In our approach, using *rarity* as a criterion for PAN performance emphasizes the background of the geographic areas that have climates dissimilar to the climates within the units of the PAN, and identifies geographic areas where the acquisition of new protected areas might have maximal impact on the climate total climate envelope protected in the PAN. This rarity metric can be assessed by the landscape distribution of climate envelope richness (Figs 2 and 3) or by the proportion of larger geographic areas (e.g. states or ecoregions) that are populated by these predicted envelopes (Table 1). Alternatively, rarity metrics can be derived to assess the relative landscape uniqueness of a park climate (e.g. the ratio of boundary area to envelope area, or the fraction of network units with climates predicted for extinction, Supplementary S2 Table). Protected areas which are climatically similar to the geographical background may face different management challenges than protected areas that protect relative climatic novelty (i.e. have a higher frequency of rarity in the network). For example, large areas of the Midwest and Great Plains have few parks of any large area, and large parks are clustered in the Southwest and Southeast (Fig 1). In many geographic regions, the climates conserved within the PAN units are relatively rare across the landscape (Figs 2 and 3). Even in the hypothetical total absence of information about species occurrences, climate envelopes could still serve as a meaningful proxy for making management decisions based on the composition and rarity of climate envelopes within a network.

### In-situ climate refugia for PANs as future quality

If we prioritize the *future quality* of protected areas, an outcome of “no change in the climate envelope area” would be an optimal outcome for an ecologically resilient protected area. This is because our estimate of the future ecological quality of some protected area will be highest when the future climate in that protected area is most similar to the climate experienced by the area under current conditions. If climate envelopes generally tend to shift with warming, a protected area where the current climate envelope is predicted to persist into future conditions may be potentially useful as core habitat for species with preferences for the climate in that particular envelope. Areas where these persisting protected area climate envelopes “stack up” may be important for assessing whether these sites are potentially suitable for assessments of the feasibility of assisted migration, or for designing dispersal corridors and other climate refugia within a PAN.

Future climate projections allow us to investigate future changes in the spatial distribution or similarity of protected area environments. Our predictions are estimates of the *future quality* of the environments protected within this PAN. In our example, we predict that the future similarity of climates in protected areas will tend to decrease across the total landscape, since the maximum total envelope richness decreases across the map (from maximum of 7 in Fig 2 to a maximum of 6 in Fig 3). This is not surprising, since 22 park climate envelopes were predicted to go extinct (Fig 4A; S2 Table) and we found no significant relationship between park area and future footprint area. Concurrent with this general reduction in climate footprint distributions, the total *occupancy* of climate envelopes with the PAN is predicted to increase as environments within the PAN become more homogeneous (Fig 5). This analysis of occupancy and quality does not consider whether novel climates might replace current climate states within the PAN. Similarly, we cannot systematically examine the relationship between *occupancy* and park area, since this relationship is confounded by the clustering of parks in geographic and climate space.

One problem facing management of species, under changing environmental conditions, will require the identification of *refugia*, locations where climates do not shift outside of the bioclimatic envelope and thus enable populations to persist. Here we have identified examples from across the United States where we predict locations within national parks that maintain climates *in situ* in the future conditions (Fig 3). In some cases, these climate refugia (i.e. persisting climate states) might not lay within the boundary of the PAN, yet still be priority areas for directing land acquisition efforts in lands under other management or administration. Climate envelope analysis is an approximation of the maximum geographic extent of the potential refugia associated with a PAN. However, land use and ownership, as well as dispersal connectivity and community composition within these refugia, will influence the actual ecological outcomes experienced by populations of plants and animals in PANs and refugia [66–67].

### Considered objections

We have offered this method as a demonstration of how the climate envelope, or resource footprint, can be a quantity useful for planning for climate change conservation in a PAN. This effort is not intended as a comprehensive analysis of the fate of the climate envelopes of units within the PAN we used in this analysis (US National Parks in the conterminous 48 states), which would require modeling these distributions more intensively across ensembles of multiple climate datasets, under current and future conditions, and various emissions scenarios. Similarly, we could have expanded our climate envelope analysis to include other types of PANs, under different administration or with varying conservation significance. These choices are myriad, but our main point is that it may be useful to consider these methods as a way to describe the background distributions of environmental features within a map, or the spatial domain of a study area.

We have argued that the climate envelope of a protected area possesses a “range-like” ontology. Models of species-environment relationships often assume that species ranges are the outcomes of particular ecological and evolutionary processes. This reasoning underlies the choice to use instances of occurrences of individuals of a species as a sample from the multivariate environmental space where individuals have positive fitness [43]. The climate envelope of a protected area has no such evolutionary history or biological signal, but it still remains true that for any map of environmental features, any particular geographic region will inherit some set of environmental features from its position and location and that neighboring sites are more likely to share features than distant sites. It might be true that we could simply divide a PAN into multiple arbitrary subunits and derive different climate envelopes for these smaller entities, but we emphasize that in the case of a PAN we are explicitly modeling lands that are under unified or centralized management. Any account of what climates or ecological features occur within a protected area or region is described in this same way, our method simply takes advantage of the “range-like” nature of a PAN unit to evaluate the composition, rarity, persistence and quality of the environmental features protected within a PAN.

It’s now easy for most researchers or protected area network managers to get climate data for the regions in which they work, but it may still be much harder to get good species distribution data or predictions for those PANs. The climate envelope idea is similar to the idea of *geodiversity* in conservation planning [68–70], in that it implicitly recognizes that PANs have abiotic characteristics that can be compared against the background of abiotic characteristics in the surrounding landscape. Where our analysis differs from these efforts is in the use of SDM techniques to explicitly predict the geographic extent of the climate occurring within a protected area and the climatic refugia associated with this PAN in future conditions. The enormous variety of lands under management in any real world setting means that PAN climate envelopes, or other predictive models of other resource footprints, could be leveraged to conserve particular biodiversity and ecological processes.

## Supporting information

S1 Table Abbreviations, locations and full names of 163 national parks in this study. (PDF)

S2 Table Climate footprint dynamics for 163 national parks. Footprints modeled under current and future climate conditions. Future climate conditions are derived from the 2050 HADCM3 emissions scenario a2a (accessed from BIOCLIM site Aug. 2011).
(PDF)
