# Supplementary material for: Species-free species distribution models describe macroecological properties of protected area networks
Source: PLoS One. 2017 Mar 16;12(3):e0173443. doi: 10.1371/journal.pone.0173443 (PMC5354291; doi:10.1371/journal.pone.0173443)
Supplement: S1 Table — (PDF) [file pone.0173443.s001.pdf]

S1 Table

Park ID	Location
ACAD	Acadia National Park, ME
AGFO	Agate Fossil Beds National Monument, NE
ALFL	Alibates Flint Quarries National Monument, TX
ALPO	Allegheny Portage Railroad National Historic Site, ME
AMIS	Amistad National Recreational Area, TX
APCO	Appomattox Courthouse National Historical Site, VA
APIS	Apostle Islands National Seashore, WI
ARCH	Arches National Park, UT
BADL	Badlands National Park, SD
BAND	Bandelier National Monument, NM
BIBE	Big Bend National Park, TX
BICA	Bighorn Canyon National Recreation Area, MT
BICY	Big Cypress National Preserve, FL
BISO	Big South Fork National River and Recreation Area, TN
BITH	Big Thicket National Preserve, TX
BLCA	Black Canyon of the Gunnison National Park, CO
BLRI	Blue Ridge Parkway, NC
BLUE	Bluestone National Scenic River, WV
BRCA	Bryce Canyon National Park, UT
BUFF	Buffalo National River, AR
CACH	Canyon de Chelly National Monument, AZ
CARE	Capitol Reef National Park, UT
CATO	Catoctin Mountain National Park, MD
CAVE	Carlsbad Caverns National Park, NM
CEBE	Cedar Creek and Belle Grove National Historical Park, VA
CEBR	Cedar Breaks National Monument, UT
CHAT	Chattahoochee River National Recreation Area, GA
CHCU	Chaco Culture National Historical Park, NM
CHIC	Chickasaw National Recreation Area, OK
CHIR	Chiricahua National Monument, AZ
CHIS	Channel Islands National Monument, CA
CHOH	Chesapeake and Ohio Canal National Historical Park, MD
CIRO	City of Rocks National Reserve, ID
COLM	Colorado National Monument, CO

COSW	Congaree National Park, SC
CRLA	Crater Lake National Park, OR
CRMO	Craters of the Moon National Monument and Preserve, ID
CUGA	Cumberland Gap National Historic Park, KY
CURE	Curecanti National Recreation Area, CO
CUVA	Cuyahoga Valley National Park, OH
DETO	Devils Tower National Monument, WY
DEVA	Death Valley National Park, NV
DEWA	Delaware Water Gap National Recreation Area, NJ
DINO	Dinosaur National Monument, CO
EFMO	Effigy Mounds National Monument, IA
ELMA	El Malpais National Monument, NM
EVER	Everglades National Park, FL
FLFO	Florissant Fossil Beds National Monument, CO
FLNI	Flight 93 National Monument, PA
FOBO	Fort Bowie National Historic Site, AZ
FOBU	Fossil Butte National Monument, WY
FONE	Fort Necessity National Battelfield, PA
FRSP	Fredericksburg and Spotsylvania National Military Park, VA
GARI	Gauley River National Recreation Area, WV
GETT	Gettysburg National Military Park, VA
GLAC	Glacier National Park, MT
GLCA	Glen Canyon National Recreation Area, AZ
GOGA	Golden Gate National Recreation Area, CA
GOSP	Golden Spike National Historic Site, UT
GRBA	Great Basin National Park, NV
GRCA	Grand Canyon National Park, AZ
GRKO	Grant-Kohrs Ranch National Historic Site, MT
GRPO	Grand Portage National Monument, MN
GRSA	Great Sand Dunes National Park and Preserve, CO
GRSM	Great Smoky Mountains National Park, TN
GRSP	Green Springs National Historic Landmark District, VA
GRTE	Grand Teton National Park, WY
GUMO	Guadalupe Mountains National Park, TX
HAFO	Hagerman Fossil Beds National Monument, ID
HOBE	Horseshoe Bend National Military Park, AL
HOCU	Hopewell Culture National Historical Park, OH
HOFR	Home of Franklin D. Roosevelt National Historic Site, NY

HOSP	Hot Springs National Park, AR
HOVE	Hovenweep National Monument, CO
ILMI	Illinois and Michigan Canal National Heritage Corridor, IL
INDU	Indiana Dunes National Lakeshore, IN
ISRO	Isle Royale National Park, MN
JELA	Jean Lafitte National Historical Park and Preserve, LA
JODA	John Day Fossil Beds National Monument, OR
JODR	John D. Rockefeller Memorial, WY
JOTR	Joshua Tree National Park, CA
KEMO	Kennesaw Mountain National Battlefield Park, GA
KEWE	Keweenaw National Historical Park, MI
KICA	Kings Canyon National Park, CA
KIMO	Kings Mountain National Military Park, SC
KNRI	Knife River Indian Villages National Historic Site, ND
LABE	Lava Beds National Monument, CA
LACH	Lake Chelan National Recreational Area, WA
LAME	Lake Mead National Recreation Area, NV
LAMR	Lake Merideth National Recreation Area, TX
LARO	Lake Roosevelt National Recreation Area, WA
LAVO	Lassen Volcanic National Park, CA
LIBI	Little Bighorn Battlefield National Monument, SD
LIRI	Little River Canyon National Preserve, AL
LOWE	Lowell National Historical Park, MA
LYJO	Lyndon B. Johnson National Historical Park, TN
MACA	Mammoth Cave National Park, KY
MANA	Manassas National Battlefield Park, VA
MEVE	Mesa Verde National Park, CO
MIMA	Minute Man National Historical Park, MA
MNRR	Missouri River National Recreational Area, MO
MOJA	Mojave National Preserve, CA
MONO	Monocacy National Battlefield, MD
MORA	Mount Ranier National Park, WA
MORR	Morristown National Historical Park, NJ
MORU	Mt. Rushmore National Memorial, SD
NABR	Natural Bridges National Monument, UT
NACC	National Mall and Memorial Parks, DC
NACE	National Capital Parks East, DC
NATR	Natchez Trace Parkway, MS

NEPE	Nez Perce National Historical Park, ID
NERI	New River Gorge National River, WV
NIOB	Niobrara National Scenic River, NE
NOCA	North Cascades National Park, WA
OBRI	Obed Wild and Scenic River, TN
OLYM	Olympic National Park, WA
ORPI	Organ Pipe Cactus National Monument, AZ
OZAR	Ozark National Scenic Riverways, MO
PAAL	Palo Alto Battlefield National Historical Park, TX
PECO	Pecos National Historical park, NM
PEFO	Petrified Forest National Park, AZ
PERI	Pea Ridge National Military Park, AR
PETE	Petersburg National Battlefield, VA
PETR	Petroglyph National Monument, NM
PIMA	Hohokam Pima National Monument, AZ
PINN	Pinnacles National Monument, CA
PIRO	Pictured Rocks National Lakeshore, MI
PORE	Point Reyes National Seashore, CA
PRWI	Prince William Forest Park, VA
REDW	Redwood National Park, CA
RICH	Richmond National Battlefield Park, VA
ROCR	Rock Creek Park, DC
ROLA	Ross Lake National Recreation Area, WA
ROMO	Rocky Mountain National Park, CO
SAAN	San Antonio Missions National Historical Park, TX
SACN	Saint Croix National Scenic Riverway, WI
SAGU	Saguaro National Park, AZ
SAMO	Santa Monica Mountains National Recreation Area, CA
SAND	Sand Creek Massacre National Historic Site, CO
SAPU	Salinas Pueblo Missions National Monument, NM
SARA	Saratoga National Historical Park, NY
SEQU	Sequoia and Kings Canyon National Park, CA
SHEN	Shenandoah National Park, VA
SHIL	Shiloh National Military Park, TN
SLBE	Sleeping Bear Dunes National Lakeshore, MI
STRI	Stones River National Battlefield, TN
SUCR	Sunset Crater Volcano National Monument, AZ
TAPR	Tallgrass Prairie National Preserve, KS

THRO	Theodore Roosevelt National Park, ND
TIMU	Timucuan Ecological and Historic Preserve, FL
UPDE	Upper Delaware Scenic and Recreational River, NY
VAFO	Valley Forge National Historical Park, PA
VICK	Vicksburg National Military Park, MS
VOYA	Voyageurs National Park, MN
WACA	Walnut Canyon National Monument, AZ
WHIS	Whiskeytown National Recreational Area, CA
WHSA	White Sands National Monument, NM
WICA	Wind Cave National Park, SD
WICR	Wilson's Creek National Battlefield, MO
WUPA	Wupatki National Monument, AZ
YELL	Yellowstone National Park, WY
YOSE	Yosemite National Park, CA
ZION	Zion National Park, UT
