# Supplementary material for: Species-free species distribution models describe macroecological properties of protected area networks
Source: PLoS One. 2017 Mar 16;12(3):e0173443. doi: 10.1371/journal.pone.0173443 (PMC5354291; doi:10.1371/journal.pone.0173443)
Supplement: S2 Table — Footprints modeled under current and future climate conditions. Future climate conditions are derived from the 2050 HADCM3 emissions scenario a2a (accessed from BIOCLIM site Aug. 2011). (PDF) [file pone.0173443.s002.pdf]

S2 Table

Park ID	Park Area (km^2)	Ecoregions	Current Footprint Area	a2a Footprint Area
ACAD	106	1	988	1697
AGFO	47	1	970	1815
ALFL	20	1	589	411216
ALPO	48	1	5288	94667
AMIS	690	1	6697	32
APCO	22	1	2724	28087
APIS	324	1	14284	0
ARCH	565	1	7951	15912
BADL	1949	1	26929	29109
BAND	276	2	49923	39368
BIBE	4426	1	42466	68667
BICA	1079	3	58213	266595
BICY	4009	1	18198	4645
BISO	949	1	20812	2228
BITH	1029	2	12628	11278
BLCA	262	2	35446	24146
BLRI	1948	1	90411	4984
BLUE	62	1	1011	0
BRCA	307	1	45526	19355
BUFF	890	2	26065	451
CACH	752	2	65918	55497
CARE	3992	3	56997	42841
CATO	64	1	2331	0
CAVE	360	2	5565	2151
CEBE	47	1	1282	1153
CEBR	54	1	3120	197
CHAT	214	1	16834	26448
CHCU	297	2	35787	26970
CHIC	109	1	21793	245701
CHIR	92	1	70869	68647
CHIS	29	1	824	44
CHOH	761	2	37330	6170
CIRO	124	1	33556	1967
COLM	180	2	13906	17798
COSW	211	1	1255	0
CRLA	1300	1	34027	18785
CRMO	3800	2	33589	32359

CUGA	243	1	7977	2351
CURE	458	2	18424	28
CUVA	349	2	8603	0
DETO	20	1	1668	114150
DEVA	20614	2	86037	97506
DEWA	582	1	25557	3995
DINO	1626	2	42387	8546
EFMO	42	1	4243	52214
ELMA	857	1	8154	1472
EVER	3492	1	10115	9339
FLFO	63	1	588	0
FLNI	32	1	997	0
FOBO	22	1	1322	206
FOBU	88	1	8725	0
FONE	21	2	1899	0
FRSP	187	1	16682	6851
GARI	157	1	10609	2021
GETT	86	2	7237	1539
GLAC	7455	2	37827	1995
GLCA	2051	2	55869	117387
GOGA	331	2	5680	3308
GOSP	68	1	4135	113822
GRBA	557	1	16386	67
GRCA	8158	3	91991	60917
GRKO	27	1	390	0
GRPO	40	1	721	0
GRSA	825	2	27495	1601
GRSM	3455	2	24937	8
GRSP	129	1	8665	3056
GRTE	2247	1	21543	37961
GUMO	589	2	13626	230
HAFO	57	1	1308	117329
HOBE	25	1	1975	93447
HOCU	38	1	6929	131571
HOFR	20	1	3190	20071
HOSP	66	1	1320	1066357
HOVE	31	1	3956	51411
ILMI	2601	2	21880	17928
INDU	233	1	20492	57237
ISRO	1009	1	8037	1998
JELA	162	2	3359	72598
JODA	165	2	34816	0

JODR	205	1	5965	27173
JOTR	4858	2	49443	59
KEMO	43	1	1307	54121
KEWE	33	1	389	1194
KICA	2975	1	27426	711
KIMO	42	1	1771	0
KNRI	30	1	736	620
LABE	365	1	3639	0
LACH	539	1	7553	14408
LAME	9877	2	99527	112707
LAMR	391	1	6730	22989
LARO	1405	1	30490	6311
LAVO	778	1	38242	10975
LIBI	21	1	623	391182
LIRI	165	1	4839	2721
LOWE	21	1	405	303399
LYJO	30	1	1858	14
MACA	396	1	5151	0
MANA	58	1	953	35570
MEVE	394	1	12792	19191
MIMA	30	1	351	216254
MNRR	899	2	32966	263895
MOJA	9574	1	41439	9022
MONO	27	1	1049	18707
MORA	1758	1	35261	4411
MORR	37	1	596	13735
MORU	23	1	455	0
NABR	71	1	2473	3081
NACC	36	1	487	116531
NACE	131	1	6479	70020
NATR	1628	3	106447	27299
NEPE	99	3	79298	27881
NERI	647	1	11748	0
NIOB	409	1	36897	171369
NOCA	3917	1	31276	11246
OBRI	167	1	6897	2798
OLYM	6636	1	34152	4870
ORPI	1814	1	13811	1520
OZAR	870	1	27511	31166
PAAL	29	1	940	34812
PECO	77	2	1887	3552
PEFO	1477	1	13899	2855

PERI	47	1	1074	0
PETE	77	2	2576	1595190
PETR	100	1	2145	699
PIMA	22	1	1769	3533
PINN	220	1	3640	851
PIRO	554	1	20550	2
PORE	28	1	682	786
PRWI	106	1	2469	0
REDW	602	2	24613	10716
RICH	59	1	1595	246318
ROCR	114	1	4111	4244
ROLA	924	1	28219	14140
ROMO	1840	1	45731	2127
SAAN	38	1	936	183963
SACN	1466	2	86739	175846
SAGU	656	2	21297	70830
SAMO	651	1	15558	11269
SAND	114	1	1911	7457
SAPU	28	1	2723	2810
SARA	42	1	1844	51687
SEQU	2382	1	27092	5743
SHEN	1151	1	44759	103
SHIL	23	1	473	2628270
SLBE	529	1	11571	0
STRI	25	1	970	810
SUCR	33	1	1289	2459
TAPR	101	1	4917	67711
THRO	629	1	21785	7405
TIMU	139	1	1697	108
UPDE	566	2	12082	0
VAFO	39	1	2035	254366
VICK	33	1	720	14548
VOYA	1584	1	4592	0
WACA	54	2	802	4437
WHIS	321	2	19079	13249
WHSA	952	1	11166	16550
WICA	249	1	43606	18150
WICR	26	1	1053	1378
WUPA	287	1	4381	121070
YELL	15055	2	89755	76989
YOSE	4703	1	50230	8084
ZION	1028	2	9803	4702
